# Trends in Pharmacological Treatment of Patients With New Onset Type 2 Diabetes: Usage Patterns in an Evolving Guideline Landscape

**DOI:** 10.1111/1753-0407.70108

**Published:** 2025-06-04

**Authors:** William Hou, Katherine R. Tuttle, Weining Shen, Andrew Reikes, Jonathan H. Watanabe

**Affiliations:** ^1^ Donald Bren School of Information and Computer Sciences University of California Irvine California USA; ^2^ Nephrology Division University of Washington School of Medicine Seattle Washington USA; ^3^ Providence Medical Research Center Providence Inland Northwest Health Spokane Washington USA; ^4^ Department of Statistics University of California Irvine California USA; ^5^ Donald Bren School of Information and Computer Sciences Irvine California USA; ^6^ Divisions of General Internal Medicine and Endocrinology, Department of Medicine University of California, Irvine School of Medicine Irvine California USA; ^7^ Department of Clinical Pharmacy University of California San Francisco San Francisco California USA

**Keywords:** antidiabetic medications, cardiovascular disease, diabetes, guidelines, insulins, metformin, real‐world data

## Abstract

**Aims:**

In patients with new onset type 2 diabetes, this study aimed to analyze glucose‐lowering medication use patterns between 2014 and 2022.

**Materials and Methods:**

This retrospective study included adults with incident type 2 diabetes in the University of California Health System between 2014 and 2022. We determined medications used within 1 year of diagnosis and evaluated statistical evidence of use pattern changes via Mann–Kendall trend tests. Four categories of high‐risk patients requiring cardio‐kidney‐metabolic protection were also evaluated in stratified analyses based on 2024 ADA guidelines.

**Results:**

Of 40 150 patients with incident type 2 diabetes, 38.5% initiated glucose‐lowering medication within 1 year. Metformin remained the most used medication from 2014 to 2022. From 2014 to 2022, usage of GLP‐1 receptor agonists and SGLT‐2 inhibitors increased exponentially. GLP‐1 receptor agonist use increased from below 2.5%–21%. While SGLT‐2 inhibitor use increased from less than 2.5%–14%. This growth coincided with a decline in sulfonylurea usage. Among high‐risk, insulin was most prevalent in those with heart failure or chronic kidney disease. However, usage of insulin declined overall in all groups. Utilization of SGLT‐2 inhibitors was particularly high in patients with prior heart failure.

**Conclusions:**

In adults with new onset type 2 diabetes, GLP‐1 receptor agonist and SGLT‐2 inhibitor utilization has markedly increased, coordinating with evolving guidelines that emphasize cardiovascular and chronic kidney disease management. However, overall adoption rates of these medications remain low based on indicated populations. Sulfonylurea use declined while metformin remains the most frequently initiated treatment.

## Graphical abstract

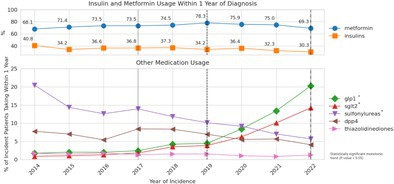

**Summary**

- Study sample included 40 150 adults with incident type 2 diabetes between 2014 and 2022.
- Utilization of sodium–glucose cotransporter 2 inhibitors and glucagon‐like peptide 1 receptor agonists has rapidly increased with the evolution of guidelines that emphasize cardio‐kidney‐metabolic risk reduction.
- Insulin, sulfonylurea, and DPP‐4 inhibitor use has declined.
- Usage of metformin and thiazolidinediones remained stable.
- While use of agents tied to improved cardio‐kidney‐metabolic risk has increased, based on guidelines, millions of US patients are likely receiving suboptimal treatment regimens.

## 1 Introduction

Greater than 1 in 10 individuals in the United States has type 2 diabetes, accruing over $400 billion in direct and indirect costs in 2022 [1]. Cardiovascular disease (CVD) is the most common cause of death in the US, with type 2 diabetes being a major risk factor for developing CVD [2]. Furthermore, individuals with this syndrome and chronic kidney disease (CKD) are at elevated risk for death and kidney failure, irrespective of CVD status [3].

Guidelines for the pharmaceutical treatment of type 2 diabetes have evolved to prioritize the management of cardio‐kidney‐metabolic comorbidities from prior foci on blood glucose management [4]. Since 2019, the American Diabetes Association has recommended sodium–glucose cotransporter 2 (SGLT‐2) inhibitors or glucagon‐like peptide 1 (GLP‐1) receptor agonists with demonstrated CVD benefit for adults with established atherosclerotic CVD (ASCVD) [5]. This recommendation was later extended to adults with established CKD and prior heart failure (HF) independent of A1C in 2020 [6]. Further guideline updates occurring in 2023 reflected the management of cardio‐kidney‐metabolic comorbidities as a key component of care rather than simply a recommendation [7]. Leading clinical organizations, including Kidney Disease: Improving Global Outcomes (KDIGO) and the American Heart Association (AHA), have prominently incorporated SGLT‐2 inhibitors and GLP‐1 receptor agonists into their respective clinical practice guidelines [8, 9].

Previous studies in established type 2 diabetes patients have demonstrated underutilization of GLP‐1 receptor agonists [10] and SGLT‐2 inhibitors in patients with established CVD and/or CKD [11] that is particularly pronounced in underserved populations [12, 13, 14]. However, there is little published research of recent trends comparing GLP‐1 receptor agonist and SGLT‐2 inhibitor usage patterns to that of other glucose‐lowering drugs, particularly following substantial guideline revisions in 2019. Prior studies also focus on established type 2 diabetes patients (“prevalent patients”) rather than patients with new onset type 2 diabetes (“incident patients”).

Consequently, our objectives were to utilize a large, current, robust, real‐world database to determine the frequency of use of each medication category in a one‐year period following the first type 2 diabetes diagnosis for each patient by year from 2014 to 2022. We then statistically evaluated medication category use trends in these patients over the study interval overall and stratified by high‐risk categories. The study period encompasses an era of substantial shifts in pharmacological treatment, transitioning from glucose‐centric approaches to cardiovascular and renal‐protective strategies. This research provides critical and timely insights into the real‐world adoption patterns of novel therapeutic agents among incident type 2 diabetes patients within a large healthcare system.

## 2 Materials and Methods

### 2.1 Data Source

This retrospective repeated, cross‐sectional study included adults with first diagnosis of type 2 diabetes from January 1, 2014 to December 31, 2022 in the University of California Health Data Warehouse (UCHDW), which captures information from all six UC Health systems (Davis, Irvine, Los Angeles, Riverside, San Diego, San Francisco). The UCHDW database was operationalized by UC Health as “nonhuman subjects research.” Analyses are institutional review board exempt [15]. This study examined electronic health record data from health care records of patients from the 5 academic medical centers and 12 hospitals with associated clinics, comprising the UC Health system. Electronic health records are connected and translated into a standardized vocabulary of clinical codes using the Observational Medical Outcomes Partnership (OMOP) version 5.4. As of May 2024, the source UCDHW dataset contained records for more than 196 million visits for more than 16 million unique patients within the UC Health system. Medical visit records extend retrospectively to 2012.

### 2.2 Study Sample

**FIGURE 1:**
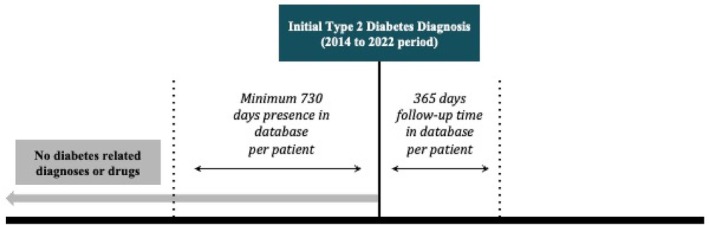
Study design diagram.

We identified adult patients (ages 18 years old and above at time of diagnosis) with incident type 2 diabetes based on preliminary type 2 diabetes diagnosis in their health record by each year of the study interval 2014–2022. We used Systematized Medical Nomenclature for Medicine–Clinical Terminology (SNOMED CT) codes 313436004 (Type 2 diabetes mellitus without complication) and 44054006 (Diabetes mellitus type 2) to identify patients with type 2 diabetes. To validate accurate type 2 diabetes incident status, study patients were required to have evidence of health record data presence in the UCHDW at least 2 years prior to first type 2 diabetes diagnosis (pre‐index period) and at least 1 year follow‐up data after type 2 diabetes diagnosis (follow‐up period) to measure medication use after new onset. Additionally, a data string search was performed to identify diseases related to or caused by “type 2 diabetes mellitus” and any patients with evidence for any such conditions before their designated study incident date were excluded (Figure 1).

We identified glucose‐lowering medications listed on the ADA 2024 Standards of Care Table 9.2: Medications for lowering glucose [16]. We determined medication use using Anatomical Therapeutic Chemical (ATC) Level 3 and 4 codes maintained by the WHO [17] and RxNorm codes maintained by the National Library of Medicine [18]. Both code sets indicate prescription events.

Insulins were identified using RxNorm codes descended from ATC code “A10A” (Insulins and Analogues). We identified Metformin using RxNorm codes descended from RxNorm code “6803” (Metformin). We identified SGLT‐2 inhibitors using RxNorm codes descended from ATC code “A10BK” (SGLT‐2 inhibitors). We identified GLP‐1 receptor agonists using RxNorm codes descended from ATC code “A10BJ” (Glucagon‐like peptide‐1 (GLP‐1) analogues) and RxNorm codes descended from RxNorm code “1 991 302” to capture the oral GLP‐1 receptor agonist, Rybelsus. We identified DPP‐4 inhibitors using RxNorm codes descended from ATC code “A10BH” (Dipeptidyl peptidase 4 (DPP‐4) inhibitors). We identified Thiazolidinediones using RxNorm codes descended from ATC code “A10BG” (Thiazolidinediones). Finally, we identified Sulfonylureas using RxNorm codes descended from ATC code “A10BB” (Sulfonylureas).

The ADA 2024 guidelines, “Use of Glucose‐Lowering Medications in the Management of Type 2 Diabetes,” were referenced to identify four high‐risk groups where CVD and CKD risk reduction is crucial: Patients with atherosclerotic CVD (ASCVD), those at high risk for ASCVD, HF, and CKD [16]. Study patients could be assigned into one or multiple groups at baseline using SNOMED codes. Logical Observation Identifiers Names and Codes (LOINC) codes were used to identify relevant laboratory measurements.

For the ASCVD group, conditions included ischemic stroke (422504002), myocardial infarction (22298006), ischemic heart disease (414545008), and disorder of coronary artery (414024009). CKD was identified using code 709044004 and included incidences due to underlying conditions. HF was identified using code 84114007.

The high risk ASCVD group included patients aged 55 or older with two or more of the following indicators: obesity (414916001, 238136002, or BMI [LOINC 39156‐5] ≥ 30) [19], hypertension (59621000), smoking status (449868002, 428041000124106, 428061000124105, 77176002), hyperlipidemia (55822004), and albuminuria [LOINC 9318‐7 ≥ 300] [20].

Baseline clinical characteristics were identified using LOINC codes 39156‐5 (BMI ratio), 8480‐6 (systolic blood pressure), and 8462‐4 (diastolic blood pressure). Measurements must have been taken on the day of the index diagnosis or in the 30 days prior. If multiple measurements were recorded, the value closest to the time of incidence was taken.

### 2.3 Graphical Indicators of Guideline Changes in Plots

Medication categorical percentage use by year over the study period were plotted for patients with incident type 2 diabetes for patients overall and separately by the four predefined high‐risk groups requiring CVD and CKD protection, established ASCVD, high ASCVD risk, HF, and CKD.

In 2017, language was added to the ADA Standards of Care to “consider [the SGLT‐2] empagliflozin or [the GLP‐1] liraglutide in patients with established CVD” [21]. Following the 2018 consensus report on management of hyperglycemia released by the ADA and European Association for the Study of Diabetes, guidelines were revised in 2019 to consider key comorbidities including ASCVD, CKD, and HF. GLP‐1 receptor agonists also became preferable to insulins as the first choice of injectable therapy [5]. Since 2022, guidelines no longer explicitly state metformin as the preferred first‐line treatment in concordance with statements recognizing alternative initial treatment approaches to metformin as acceptable [22]. To indicate these material changes to ADA guidelines for type 2 diabetes treatment over the study period, three vertical lines mark were added to all use plots at years 2017, 2019, and 2022.

### 2.4 Statistical Analysis

Annual percentage use of type 2 diabetes medication by category within the first year following diagnosis was determined for the years 2014–2022. The annual percentage response variable was defined as the proportion of patients using a specific glucose‐lowering medication category out of the total number of incident patients receiving any glucose‐lowering medication in that incident year. The *x*‐axis was segmented into intervals representing the year of diagnosis for each patient.

The analysis was repeated stratifying by each of the four predefined high‐risk groups requiring CVD and CKD protection, established ASCVD, high ASCVD risk, HF, and CKD, to examine trends specific to these subgroups.

The Mann–Kendall Trend Test [23] was used to determine statistical evidence of monotonic directional change in percentage over time for each of the glucose‐lowering medication categories used within 1 year of type 2 diabetes diagnosis. All statistical analyses were performed using Python, version 3.8, using the pyMannKendall package (Python Software Foundation, Beaverton, OR) on a protected Databricks platform workspace (Databricks, San Francisco, CA) with an *α* = 0.05 significance level applied for all statistical hypothesis tests.

## 3 Results

The study sample included 40 150 adults with incident type 2 diabetes between 2014 and 2022. The mean age was 61.1 years old, and 51% were women. The sample was 53.9% White, and 19.1% were Hispanic or Latino. Of the study sample, 38.5% initiated any glucose‐lowering medication within 1 year of incidence. A majority of the sample (54.0%) fell into high‐risk categories requiring cardio‐kidney‐metabolic protection. The mean body mass index (BMI) was in the obese range (BMI = 30.3) and mean blood pressure (systolic blood pressure = 131.3) was indicative of stage 1 hypertension (Table 1).

**TABLE 1:** Patient characteristics at time of new onset type 2 diabetes diagnosis (2014 to 2022).

Variable	Total (*N* = 40 150)
Age (mean years)	61.10
Age (median years)	62
Age (std years)	14.19
Glucose‐lowering medication within 1 year	15 474 (38.5%)
Glucose‐lowering medication ever	24 865 (61.9%)
Established ASCVD	6923 (17.2%)
Established CKD	6008 (15.0%)
Established HF	3630 (9.0%)
High ASCVD Risk	16 647 (41.5%)
Requiring cardio‐kidney‐metabolic protection	21 664 (54.0%)
Clinical characteristic (std.)	
Baseline diastolic BP (*n* = 30 466)	75.9 (±12.5)
Baseline systolic BP (*n* = 30 466)	131.3 (±18.8)
Baseline BMI (*n* = 27 565)	30.3 (±6.5)
Gender	
Women	20 466 (51.0%)
Men	19 675 (49.0%)
Ethnicity	
Not Hispanic or Latino	29 905 (74.5%)
Hispanic or Latino	7676 (19.1%)
Unknown	2569 (6.4%)
Race	
White	21 620 (53.9%)
Asian	6201 (15.4%)
Other	5198 (13.0%)
Black or African American	3593 (9.0%)
Unknown	2931 (7.3%)
Native Hawaiian or other Pacific Islander	379 (0.9%)
American Indian or Alaska Native	228 (0.6%)

### 3.1 Glucose‐Lowering Medication Use Trends

**FIGURE 2:**
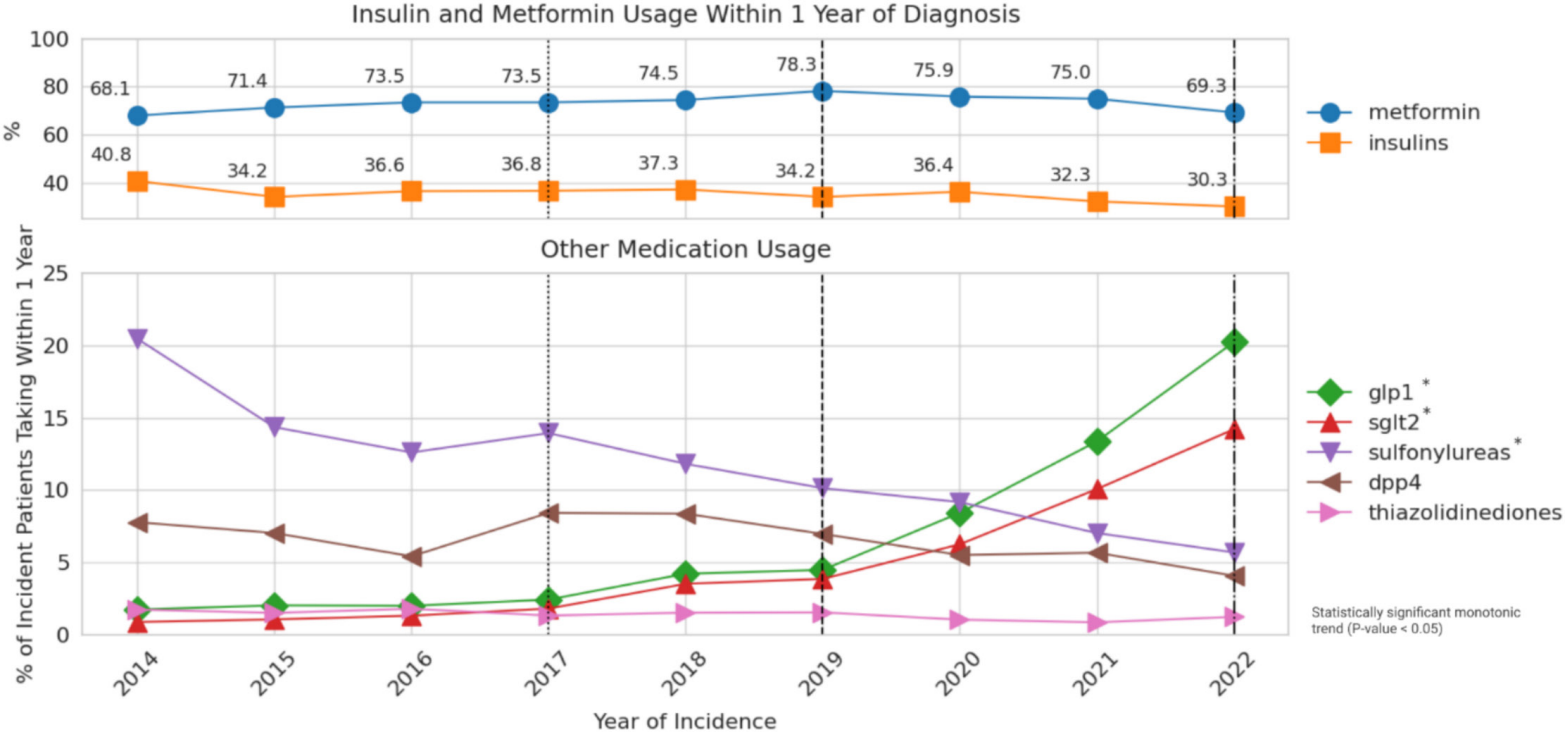
Medication Usage within 1 year of Type 2 Diabetes Diagnosis Over Time (2014 to 2022). (Vertical hatched lines indicate material changes to ADA guidelines for type 2 diabetes treatment over the study period).

Throughout the 2014–2022 study period, metformin was consistently the most prescribed medication in incident patients with type 2 diabetes, varying between a low of 68.1% (2014) to a maximum of 78.3% (2019). The variation in metformin use was not statistically significant based on the trend test (*p* = 0.12). Overall, insulins remained the second‐most utilized medication throughout the study period. While usage dropped from 40.8% in 2014 to 30.3% in 2022, the decrease was not significant based on the trend test (*p* = 0.07). The GLP‐1 receptor agonist and SGLT‐2 inhibitor categories both demonstrated evidence of statistically significant, increasing usage over time in incident patients with type 2 diabetes. Usage of GLP‐1 receptor agonists grew from 1.7% of incident patients in 2014 to 20.2% in 2022 (*p* < 0.01). Usage of SGLT‐2 inhibitors grew from 0.9% to 14.2% (*p* < 0.01) in the same 9‐year period. The rate of adoption for both medication categories accelerated significantly after 2017 and 2019. By 2021, GLP‐1 receptor agonists and SGLT‐2 inhibitors had eclipsed all other medication categories except for metformin and insulins to become the third and fourth most frequently utilized medications at 20.2% and 14.2%, respectively, with continued increase observed at the end of the study period in 2022 (Figure 2).

In 2014, sulfonylureas were the third most prescribed glucose‐lowering medication category, used in 20.5% of incident patients with type 2 diabetes taking medication. However, sulfonylurea utilization dropped every year throughout the study period to 5.7% in 2022 (*p* < 0.01). In 2014, at 7.8%, DPP‐4 inhibitors were more frequently prescribed than GLP‐1 receptor agonists and SGLT‐2 inhibitors. However, the use percentage remained relatively stable over the study period and demonstrated no statistically significant trend (*p* = 0.12). By 2020, both GLP‐1 receptor agonists and SGLT‐2 inhibitors were more commonly prescribed in incident patients with type 2 diabetes than DPP‐4 inhibitors. Throughout the study interval, thiazolidinediones were the least frequently used and remained below 5% of use (Figure 2).

### 3.2 Drug Use Trends Among High‐Risk Patients Requiring CVD and CKD Protection

Among study patients, 21 664 (54.0%) had a history of established ASCVD, CKD, HF, or were at high risk for ASCVD. Under 2024 ADA guidelines, adults with previous HF should begin treatment with an SGLT‐2 inhibitor with proven benefit. Adults with CKD, ASCVD, or high ASCVD risk should incorporate GLP‐1 receptor agonists or SGLT‐2 inhibitors.

**FIGURE 3:**
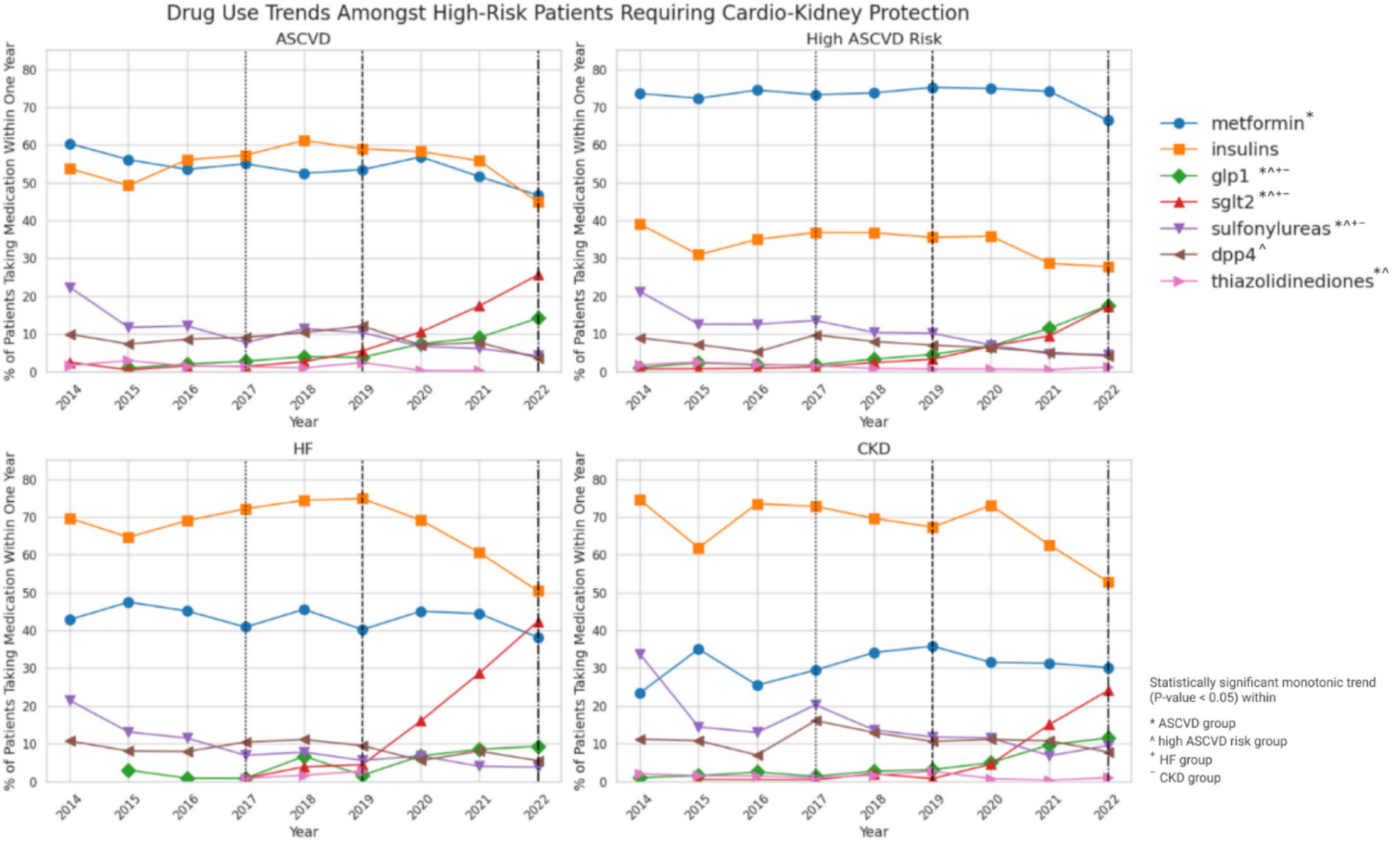
Medication usage within 1 year of type 2 diabetes diagnosis over time by high‐risk category (2014–2022). (Vertical hatched lines indicate material changes to ADA guidelines for type 2 diabetes treatment over the study period).

Across all four high‐risk categories analyzed, SGLT‐2 inhibitors and GLP‐1 receptor agonists demonstrated statistically significant growth in utilization over the study period, with each category below 10% in 2019 growing markedly thereafter (*p* < 0.01). The SGLT‐2 inhibitors increased from below 10% in 2019 to 25.06%, 17.3%, 42.4%, and 24.1% for ASCVD, high ASCVD risk, HF, and CKD patients, respectively, in 2022 (Figure 3).

For the GLP‐1 receptor agonists, compared to the SGLT‐2 inhibitors, the increase was significant but less pronounced for the four high‐risk groups. With utilization below 10% in 2019, the percentage of users rose to 15.2%, 17.5%, 9.3%, and 11.5% for ASCVD, high ASCVD risk, HF, and CKD patients in 2022, respectively (*p* < 0.01 for all four groups). While GLP‐1 receptor agonists and SGLT‐2 inhibitors both demonstrated similar increases in utilization for patients with high ASCVD risk, SGLT‐2 inhibitors were more commonly used across incident patients of other risk categories. The preponderance of SGLT‐2 inhibitor use was most evident in the HF risk category, with nearly half (42.4%) of incident type 2 diabetes patients in 2022 who had initiated any medication using a SGLT‐2 inhibitor within 1 year (Figure 3).

Sulfonylurea usage decreased in patients in all high‐risk categories (*p* < 0.01 for all four groups). Use of DPP‐4 inhibitors decreased in incident patients over time in those with high ASCVD risk (*p* < 0.05), but did not exhibit a statistically significant monotonic trend in patients with ASCVD (*p*‐value 0.47) and HF (*p* = 0.11) (Figure 3).

Among patients with HF or CKD, insulin was the most prevalent medication throughout the study period, exceeding metformin. Trend tests in insulin use were non‐significant for both groups with *p* > 0.05. Despite an increase in usage within the HF group between 2014 and 2018, insulin use began to decline markedly starting in 2019. For patients with HF, insulin use decreased from 69.6% in 2014 to 50.4% in 2022. In patients with CKD, usage fell from 74.5.0% in 2014 to 52.9% in 2022 (Figure 3).

In patients with ASCVD, both insulin and metformin were utilized at similar rates during the study period. While each was used in more than 50% of incident patients in 2014, usage declined to below half for each by 2022, with metformin reduction statistically significant via trend test (*p* < 0.01) Insulin use oscillated in patients with ASCVD during the study period in each of the high‐risk groups from 2014 to 2022 study period consistent with a non‐significant trend test (Figure 3).

## 4 Discussion

Using a current, large health‐system database, we completed a study analyzing new use of glucose‐lowering medications in adults with incident type 2 diabetes in the period between 2014 and 2022. Utilization of SGLT‐2 inhibitors and GLP‐1 receptor agonists has rapidly increased in parallel with the evolution of guidelines that emphasize reduction of cardio‐kidney‐metabolic risk. Increase in SGLT‐2 inhibitor usage was particularly evident in patients with prior HF.

However, despite profound increased use in recent years, usage of GLP‐1 receptor agonists and SGLT‐2 inhibitors within the first year of diagnosis remained lower than expected based on recent guidelines. In 2022, fewer than one in five patients with new onset type 2 diabetes taking a glucose‐lowering medication utilized GLP‐1 receptor agonists or SGLT‐2 inhibitors in the year following their diagnosis. This underutilization poses clinical concerns regarding optimal treatment given that most patients in the study sample experienced high cardio‐kidney‐metabolic risk and underscores the need to increase awareness of guidelines and recent evidence of clinical benefit. Insufficient insurance coverage, clinician unfamiliarity, and financial hardships all pose significant barriers to optimal use [24] that may be further compounded by the increase in cost burden as adults transition from commercial health plans into Medicare in a rapidly aging US [25].

The discrepancy this analysis has elucidated between evidence‐based guidelines and real‐world practice is a cause for concern that portends a high burden of disease attributable to cardiovascular and kidney complications if use rates do not further accelerate. More than 460 million people globally have type 2 diabetes, with studies suggesting the presence of CVD in more than 32% of cases [26, 27]. Within the United States, estimates are as high as 45.2% [28] with the prevalence of CKD nearing 40% [29]. CVD is a major cause of mortality in type 2 diabetes patients [26, 30] and is associated with a higher risk of HF [31]. The leading cause of CKD [32] is type 2 diabetes, with a long established link between CKD presence and higher mortality [3, 33].

Consistent with prior studies, metformin remained the most frequently initiated medication overall [11]. However, among the high‐risk groups, we observed greater utilization of insulin in patients with HF or CKD that likely stems from prior concerns about metformin in these populations. A black box warning for lactic acidosis has been particularly relevant for patients with kidney dysfunction [34]. Similar apprehension exists among populations with prior HF [35].

It will remain important to observe whether metformin remains dominant in the years to come as CVD and CKD comorbidities become more prevalent in the United States [36] and guidelines recognize the initiation of pharmacological treatment with drugs other than metformin. As suggested by this analysis, changes in clinical practice guidelines could be associated with changes in glucose‐lowering medication utilization.

As anticipated from meta‐analyses of cardiovascular outcome trials [37], we observed a greater utilization of SGLT‐2 inhibitors compared to GLP‐1 receptor agonists in patients with prior HF. Conversely, GLP‐1 receptor agonist utilization was higher overall, perhaps due to widespread interest in the weight‐loss benefits of semaglutide and evidence for ASCVD risk reduction [38]. However, SGLT‐2 inhibitors have also been linked to weight reduction [39], and the heterogeneity in weight‐loss effectiveness between these drug classes remains an active area of research [40] which ultimately could further enhance SGLT‐2 inhibitor usage.

This study has several strengths. Analyses of real‐world use patterns over time of all new use diabetes medications by category in patients with new onset type 2 diabetes have not been performed in a large, current, health system database. Our study data incorporated 9 years of health records, including those diagnosed in 2022, concurrent with an era of important evidence‐driven changes in clinical guidance from the American Diabetes Association.

Limitations of this study include that while the UCHDW database reflects the diverse population of California that includes a large proportion of minority sub‐populations, these demographics may affect the generalizability of the findings to the broader United States population. Additionally, while our inclusion criteria ensured by design that the study population consisted of patients with incident type 2 diabetes with continuous follow‐up prior to and following the diagnosis of type 2 diabetes, it may limit the capture of patterns of care in patients that receive transient care from multiple systems.

Among adults with incident type 2 diabetes, utilization of GLP‐1 receptor agonists and SGLT‐2 inhibitors increased in parallel with evolving guidelines that prioritized the management of CVD and CKD comorbidities. However, in the context of the majority prevalence of high‐risk conditions for patients with incident type 2 diabetes, overall utilization remained low. Metformin was the most frequently initiated medication overall, but may experience future decline correlated with guideline‐concordant prescribing. Our study underscores the need to better understand the barriers impeding widespread access to guideline‐based treatment. Furthermore, it stresses the importance of evaluating potential solutions to overcome these obstacles, an area where more research is urgently needed. Future research will include additional follow‐up of population‐level data to analyze the influence of the recent 2023 ADA Updated Guidelines. Comprehensive, comparative evaluation of long‐term, real‐world follow‐up for patients at high risk tied to initial treatment and outcomes will also be necessary.

## Author Contributions

J.H.W., W.H., K.R.T., and W.S. designed the study and reviewed and edited the manuscript. W.H. and J.H.W. wrote and edited the manuscript and researched the data. A.R. reviewed and edited the manuscript. All authors read and approved the final manuscript.

## Ethics Statement

As described in Methods section, The University of California Health Data Warehouse database was operationalized by UC Health as “nonhuman subjects research.” Analyses are institutional review board exempt.

## Conflicts of Interest

K.R.T. is supported by NIH research grants R01MD014712, U2CDK114886, UL1TR002319, U54DK083912, U01DK100846, OT2HL161847, UM1AI109568, OT2OD032581, and CDC project numbers 75D301‐21‐P‐12254 and 75D301‐23‐C‐18264. She has also received investigator‐initiated grant support from Travere Therapeutics Inc., Bayer, and the Doris Duke Charitable Foundation. She reports consultancy fees from AstraZeneca, Boehringer Ingelheim, Eli Lilly, Novo Nordisk, Travere Therapeutics Inc., and Pfizer, and speaker fees from Novo Nordisk. W.H., W.S., and A.R. declare no conflicts of interest or financial relationships. J.H.W. has received funding support from the California Health Benefits Review Program and a UC Irvine Health Affairs Strategic Funding grant to examine incretin mimetic use. J.H.W. serves on the Forum on Drug, Discovery, Development, and Translation of the National Academies of Sciences, Engineering, and Medicine.
